# The Utilization of Chicken Egg White Waste-Modified Nanofiber Membrane for Anionic Dye Removal in Batch and Flow Systems: Comprehensive Investigations into Equilibrium, Kinetics, and Breakthrough Curve

**DOI:** 10.3390/membranes14060128

**Published:** 2024-06-03

**Authors:** Yun-Rou Chen, Dinh Thi Hong Thanh, Quynh Thi Phuong Tran, Bing-Lan Liu, Penjit Srinophakun, Chen-Yaw Chiu, Kuei-Hsiang Chen, Yu-Kaung Chang

**Affiliations:** 1 Department of Chemical Engineering, Graduate School of Biochemical Engineering, Ming Chi University of Technology, New Taipei City 243303, Taiwan; m09138210@mail2.mcut.edu.tw (Y.-R.C.); f09138851@o365.mcut.edu.tw (D.T.H.T.); cychiu@mail.mcut.edu.tw (C.-Y.C.); 2 Faculty of Environment and Labour Safety, Ton Duc Thang University, Ho Chi Minh City 70880, Vietnam; tranthiphuongquynh@tdtu.edu.vn; 3 Department of Applied Chemistry, Chaoyang University of Technology, Taichung 413310, Taiwan; binglan@cyut.edu.tw; 4 Department of Chemical Engineering, Kasetsart University, 50 Ngamwongwan Road, Chatuchak, Bangkok 10900, Thailand; fengpjs@ku.ac.th; 5 Department of Chemical Engineering and Materials Science, Yuan Ze University, Zhongli Dist., Taoyuan City 320315, Taiwan

**Keywords:** acid orange 7, ion-exchange nanofiber membrane, waste egg white proteins, dye removal, batch and flow systems

## Abstract

This study investigated the use of chicken egg white (CEW) waste immobilized on weak acidic nanofiber membranes for removing the anionic acid orange 7 (AO7) dye in batch and continuous flow modes. Different experiments were conducted to evaluate the effectiveness of CEW-modified nanofiber membranes for AO7 removal, focusing on CEW immobilization conditions, adsorption kinetics, and thermodynamics. The CEW-modified nanofiber membrane (namely NM-COOH-CEW) exhibited a maximum AO7 adsorption capacity of 589.11 mg/g within approximately 30 min. The Freundlich isotherm model best represented the equilibrium adsorption data, while the adsorption kinetics followed a pseudo-second-order rate model. Breakthrough curve analysis using the Thomas model and the bed depth service time (BDST) model showed that the BDST model accurately described the curve, with an error percentage under 5%. To investigate AO7 elution efficiency, different concentrations of organic solvents or salts were tested as eluents. The NM-COOH-CEW nanofiber membrane exhibited promising performance as an effective adsorbent for removing AO7 dye from contaminated water.

## 1. Introduction

Dyes present significant environmental challenges due to their toxicity, resistance to natural degradation, and impact on water bodies [1,2]. Industrial dyes often contain harmful chemicals that endanger humans and aquatic life. Their complex structures make them difficult to break down naturally, leading to persistent pollution in water and soil [3,4]. When dyes dissolve in water, they cause intense coloration, reducing light penetration and affecting photosynthesis and aquatic plant growth. Additionally, dissolved dyes can form toxic breakdown products, especially under sunlight, posing risks to aquatic life and persisting in the environment for long periods. Effective treatment strategies are crucial to mitigate the impacts of dye pollution [5,6,7].

There are several ways to treat the problem of dyes in wastewater [1,2,5], including physical, chemical, and biological methods. Adsorption is a popular method to remove dyes because it is highly effective [5]. Carbon-based materials, metal oxides, and polymer-based adsorbents are commonly used due to their high surface area and ability to adsorb dyes [1,2,5]. Biological treatments like microbial degradation and phytoremediation help break down dyes into less harmful substances. Chemical methods such as oxidation or advanced oxidation processes further degrade dye molecules into non-toxic substances. However, dye removal techniques vary in effectiveness and have specific advantages and limitations. Adsorption methods, such as those using activated carbon- or polymer-based adsorbents, offer efficient removal and are cost-effective for smaller applications but can be influenced by environmental conditions and may require the regeneration of used adsorbents [2,8]. Biological treatments, like microbial degradation or phytoremediation, use natural processes to break down dyes, which is environmentally friendly but can be slow and dependent on specific conditions for optimal performance [9]. Chemical treatments, such as oxidation processes, effectively degrade dyes into less harmful compounds but may involve hazardous chemicals and require significant energy [10]. Membrane filtration, including ultrafiltration and reverse osmosis, physically removes dyes through a barrier, producing high-quality treated water but with higher operational costs and potential membrane fouling issues [11]. Each method has unique pros and cons, and the choice depends on factors like dye type, wastewater characteristics, treatment goals, and available resources. Combining these methods is often necessary for comprehensive dye removal and reducing environmental impact.

Nanoparticles and nanofibers are versatile materials with unique benefits [12,13,14]. Nanoparticles have a large surface area for efficient adsorption and catalytic reactions. They can be made from various materials and are small enough to move easily in solutions, making them ideal for drug delivery and catalysis [15,16]. In contrast, nanofibers offer an even larger surface area than nanoparticles, making them excellent for filtration and adsorption. They are strong and can withstand mechanical stress, with customizable surface chemistry and pore sizes to target specific molecules. Nanofibers are used in air and water filtration, tissue engineering, drug delivery, and protective clothing due to their exceptional properties.

In this study, polyacrylonitrile (PAN) nanofibers were chosen for membrane modification due to their favorable properties. PAN nanofibers possess a blend of structural, chemical, and mechanical characteristics that make them ideal for various applications, especially where high surface area, customized surface chemistry, and mechanical resilience are needed. They are chemically stable and exhibit strong mechanical strength, making them versatile for different applications. PAN nanofiber membranes can easily be modified with specific groups like carboxylic acid (-COOH), amino (-NH_2_), or hydroxyl (-OH) to enhance dye adsorption properties. Their high porosity and interconnected pore structure promote rapid mass transfer and diffusion of dye molecules, enhancing treatment efficiency. Moreover, nanofibers are durable and can withstand challenging conditions, making them suitable for diverse wastewater treatment applications [17,18,19,20,21,22,23,24,25,26,27,28,29,30,31].

In recent years, protein-immobilized nanofiber membranes have been used to treat dyes or heavy metals from wastewater and even biological wastes (e.g., *E. coli*). This is due to the presence of diverse functional groups (e.g., polar, non-polar, and charge groups) on immobilized proteins [17,18,22,25]. In our previous work [17,18], we focused on modifying weakly acidic nanofiber membranes (NM-COOH) with waste proteins such as bovine serum albumin (BSA) or chicken egg white (CEW) for dye removal from water. We chose cationic toluidine blue O (TBO) and anionic acid orange 7 (AO7) dyes commonly used in textile dyeing. Previous studies showed that nanofiber membranes with carboxylic acid groups (NM-COOH-BSA and NM-COOH-CEW) had high adsorption capacities for TBO, reaching 434.9 mg/g and 546.3 mg/g, respectively. Additionally, we developed a dual-functional NM-COOH-CS-CEW protein nanofiber membrane that can treat both AO7 and TBO simultaneously, with adsorption capacities of 317.3 mg/g for TBO at pH 10 and 3.4 mg/g for AO7 at pH 2 [22]. These dye removal experiments using PAN-based nanofiber membranes were conducted in batch operations.

This study used chicken egg white (CEW) protein as the immobilized substance. CEW protein, a complex mixture found in egg white, has unique properties suitable for various applications like adsorption studies and biomaterial development [17,18]. The three main proteins in CEW constitute about 76% of its total protein content: ovalbumin (54%), conalbumin (12%), and ovomucoid (11%) [32]. Considering the composition, structure, and functional properties of chicken protein is crucial when designing experiments and interpreting results.

Previously, the NM-COOH-CEW nanofiber membrane effectively removed cationic toluidine blue O (TBO) dye [18], but its ability to remove anionic acid orange 7 (AO7) dye was unexplored. This study aimed to comprehensively assess AO7 adsorption using the NM-COOH-CEW nanofiber membrane, investigating parameters such as CEW immobilization time, concentration during coupling, contact time, pH, temperature, and initial AO7 concentration. Equilibrium and kinetic models were used to analyze AO7 adsorption behavior, with desorption and membrane reusability studies. The Thomas and bed depth service time (BDST) models were used to fit breakthrough curves in column studies, providing insight into AO7 adsorption dynamics in flow systems for dye removal applications.

## 2. Materials and Methods

### 2.1. Materials

All materials and equipment were sourced in accordance with previously published work [17,18]. Polyacrylonitrile (PAN) with a molecular weight of 120,000 g/mol, composed of 93% CH_2_=CHCN acrylonitrile and 7% CH_3_CO_2_CH=CH_2_ vinyl acetate, was supplied by Fortune Industries International, Inc. (New Taipei City, Taiwan). Polyethylene terephthalate (PET) nonwoven fabric (basis mass 15 g per m^2^, thickness 90 μm, fiber diameter 300–500 μm) was also acquired from Fortune Industries International, Inc. (New Taipei City, Taiwan). The electrospinning apparatus was purchased from Falco Tech Enterprise Co., Ltd. (New Taipei City, Taiwan). Acid orange 7 (AO7, Figure S1) and Toluidine blue (TBO, Figure S1) dyes with a molecular weight of 350.32 g/mol and 305.83 g/mol, respectively, 1-ethyl-3-(3-dimethylaminopropyl) carbodiimide (EDC), N-hydroxysuccinimide (NHS), ethylene glycol (EG), and other chemicals were purchased from Sigma-Aldrich (St. Louis, MO, USA). The chicken egg white solution utilized in this study was derived from chicken egg white (CEW) powder obtained from Sigma-Aldrich (Saint Louis, MO, USA). Initially, this CEW solution served as a crucial component in the process of purifying lysozyme, a common protein found in egg whites with antibacterial properties. Following the purification procedure, a residual amount of the CEW solution remained, which was deemed expired and consequently considered waste. Therefore, to prevent unnecessary wastage and to make efficient use of resources, the expired CEW solution was repurposed for the experiments conducted in this work.

### 2.2. Preparation of CEW-Modified Nanofiber Membrane

The CEW-modified nanofiber membrane was prepared using protocols outlined in prior studies [17,18]. The physical and chemical properties of these nanofibers are detailed in Supplementary Information (Section S1).

### 2.3. Operating Parameters on CEW Immobilization and AO7 Capture

A schematic depicting the capture of AO7 by NM-COOH-CEW is shown in Figure S2. The experimental procedures were performed according to the protocols outlined in our previous publications [17,18,22]. 

### 2.4. Kinetic and Equilibrium Isotherm Studies

A single NM-COOH-CEW nanofiber piece measuring 4.91 cm^2^ was placed in a solution containing AO7 dye at 1 mg/mL concentration. The mixture was stirred at 150 rpm and 25 °C for 0–3 h to study dye adsorption kinetics. For isothermal adsorption experiments, 50 mL flasks with AO7 concentrations ranging from 1 to 3 mg/mL at pH 2 were used to incubate NM-COOH-CEW nanofiber membranes. The dye solutions were mixed with the nanofiber membranes at 150 rpm and 25 °C, and after three hours, the remaining dye in the solution was measured. The dye adsorption capacity of the nanofiber membrane at equilibrium was then determined. The AO7 adsorption capacity of the nanofiber membrane can be described as either milligrams of AO7 per gram of membrane (mg AO7/g membrane) or micromoles of AO7 per gram of membrane (µmol AO7/g membrane).

Four kinetic rate models were applied to study dye adsorption onto nanofiber membranes [33,34]. The pseudo-first-order model (Equation (1)) suggests adsorbate transfer from solution to surface as the rate-limiting step. The pseudo-second-order model (Equation (2)) proposes chemisorption between the adsorbent and adsorbate as rate-limiting. The Elovich model (Equation (3)) suggests decreasing active site availability with surface occupation. Lastly, the intra-membrane diffusion model (Equation (4)) describes dye diffusion within the membrane as controlling adsorption.

(1) $$\ln(q_{1}^{*}-q_{t})=\ln q_{1}^{*}-k_{1}t\Leftrightarrow \ln\left(\frac{q_{1}^{*}-q_{t}}{q_{1}^{*}}\right)=-k_{1}t$$

(2) $$\left(\frac{t}{q_{t}}\right)=\left(\frac{1}{k_{2}\times {\left(q_{2}^{*}\right)}^{2}}\right)-\left(\frac{1}{q_{2}^{*}}\right)t$$

(3) $$q_{t}=\frac{1}{\beta }ln\left(\alpha \beta \right)+\frac{1}{\beta }lnt=\frac{1}{\beta }\ln\left(\alpha \beta t\right)$$

(4) $$q_{t}=k_{i}\times t^{0.5}+I$$

where $q_{1}^{*}$, $q_{2}^{*}$, and $q_{t}$ (mg/g) are the removal capacity for AO7 dye at equilibrium and contact time *t* (min), respectively; *k*_1_ (1/min), *k*_2_ (g/mg·min), and *k_i_* (mg/g·min^0.5^) are the pseudo-first-order kinetics, pseudo-second-order kinetics, and intra-particle diffusion’s rate constant, respectively. α (mg/g·min) and β (g/mg) are the initial sorption rate and constant related to the extent of surface coverage and activation energy for chemisorption in the Elovich model, respectively. *I* (mg/g) is the intercept, which represents the boundary layer effect.

Three equilibrium isotherm models were used to analyze dye adsorption by the nanofiber membrane: Langmuir, Freundlich, and Temkin. The Langmuir model describes monolayer adsorption on a uniform surface with fixed identical adsorption sites. The Freundlich model assumes multilayer adsorption on a varied surface with different adsorption energies. The Temkin model accounts for uniform adsorbate–adsorbent interactions with a distributed heat of adsorption across the surface. These models were applied to fit experimental data [34,35].

(5) $$\left(\frac{C^{*}}{q^{*}}\right)=\left(\frac{1}{q_{max}\times K_{L}}\right)+\left(\frac{C^{*}}{q_{max}}\right)$$

(6) $$\ln q^{*}=\ln K_{F}+\frac{1}{n}\ln C^{*}$$

(7) $$q^{*}=\frac{RT}{b}\ln\left(K_{T}\right)+\frac{RT}{b}\ln\left(C^{*}\right)\Leftrightarrow q^{*}=\frac{RT}{b}\ln\left(K_{T}\times C^{*}\right)$$

where q* (mg/g) and *q_max_* (mg/g) refer to the equilibrium and maximum removal capacities for AO7 dye in batch mode, respectively. C* (mg/mL) represents the equilibrium concentration of AO7 dye in the aqueous solution. *K_L_* (mL/mg), *K_F_* (mg/g), and *K_T_* (mL/mg) are the Langmuir, Freundlich, and Temkin constants associated with adsorption intensity, respectively. The gas constant *R* is 8.314 J/(mol·K), *T* denotes the absolute temperature (K), and *b* (J/mol) is a constant related to the adsorption energy.

The van’t Hoff equation relates equilibrium constants to temperature [34,35], enabling the determination of thermodynamic parameters like enthalpy change (Δ*H*°) based on experimental data at different temperatures. It is used to study how reactions respond to temperature variations, providing insights into reaction energetics and spontaneity. Equation (8) is a common linear form of the van’t Hoff equation, widely used to calculate changes in enthalpy (Δ*H*°) and entropy (Δ*S*°) during adsorption processes.

(8) $$\ln K_{L}=\left(\frac{\Delta S^{{}^{\circ}}}{R}\right)-\left(\frac{\Delta H^{{}^{\circ}}}{R}\right)\times \frac{1}{T}$$

### 2.5. Desorption Studies

For the dye elution experiments, various salts (0.25–1.0 M NaCl, (NH_4_)_2_SO_4_, and KSCN), as well as organic solvents (e.g., 10–50% *v/v* C_2_H_5_OH, (CH₂OH)₂, or C_3_H_8_O_3_), were utilized, following the established procedures outlined in our previous works [17,18,22]. The amount of CEW desorbed was divided by the initial amount of immobilized CEW on the membrane to calculate the CEW desorption yield.

### 2.6. Removal of AO7 Dye in Flow Process

The continuous flow adsorption experiments were carried out using a membrane reactor containing a single piece of membrane weighing approximately 0.015 g. The membrane did not include a PET supporting layer and had an effective surface area (*A_M_*) of 3.70 cm^2^ and a volume (*V_M_*) of 4.26 × 10⁻^2^ cm^3^, with a surface porosity (*ε*) of 83.65%, as detailed in a previous study [25]. The flow system was loaded with a solution of AO7 dye at a concentration of 1 mg/mL at pH 2 and a constant flow rate of 1.0 mL/min was maintained throughout the experiment. At designated intervals, 20 mL samples of the AO7 dye solution were collected for analysis after passing through the reactor.

### 2.7. Breakthrough Parameter Analysis

The permeation flux (*J*, mL/(cm^2^·min)) for the flow process was determined using Equation (9) [25,36].

(9) $$J=\left(\frac{V}{A_{M}\times t}\right)$$

The residence time in the membrane, *τ* (min), was determined using Equation (10) [25,37].

(10) $$\tau =\frac{\varepsilon \times V_{M}}{F}$$

where the membrane volume (*V_M_*) is approximately 3.39 × 10^−2^ mL and the porosity of the membrane porosity (*ε*) is approximately 83.67%.

During the experiment, when the outlet CEW concentration approached the inlet concentration (i.e., $\frac{C}{C_{0}}=1)$, this ratio represented the total binding capacity or equilibrium dynamic binding capacity (EBC) of the NM-COOH-CEW nanofiber membrane. The dynamic binding capacity at the 10% breakthrough point (DBC) assesses the dynamic adsorption performance. The volume and time collected at the breakthrough point are denoted as *V_b_* (mL) and *t_b_*, respectively. The bed volume (*BV*, Equation (11)) indicates the feed volume ratio at the 10% breakthrough (*V_b_*) to the membrane bed volume.

(11) $$BV=\left(\frac{V_{b}}{V_{M}}\right)$$

The productivity (*P*) was determined by dividing the DBC by the tb using Equation (12) [38].

(12) $$P=\left(\frac{DBC}{t_{b}}\right)$$

Membrane bed utilization (*MBU*, %) was defined as the ratio of the DBC to EBC, as shown in Equation (13) [38].

(13) $$MBU(\%)=\left(\frac{DBC}{EBC}\right)\times 100\%$$

Additionally, the membrane adsorber exhaustion rate (MAER, Equation (14)) [23,25] was used, representing the membrane mass required (g) per unit CEW adsorbed (mL) at the 10% breakthrough.

(14) $$MAER=\left(\frac{W_{M}}{V_{b}}\right)$$

The length of the mass transfer zone (*HMTZ*, Equation (15)) indicated the CEW adsorption region within the membrane bed [23,39]. It represented the region where the outlet concentration reached 10–90% of the inlet concentration. A reduced *HMTZ* indicated enhanced adsorption performance [25].

(15) $$HMTZ=Z\left(\frac{t_{90\%}-t_{10\%}}{t_{90\%}}\right)$$

where *t*_90%_ represents the time to reach 90% breakthrough (min), *t*_10%_ represents the time to reach 10% breakthrough (min), and Z represents the membrane-bed length (cm).

### 2.8. Breakthrough Curve Modeling

Utilizing dynamic models such as the Thomas model and the bed depth service time (BDST) model enables the prediction of the breakthrough curve and the estimation of the maximum binding capacity.

#### 2.8.1. Thomas Model

The Thomas model, assuming negligible axial dispersion and adhering to second-order reversible kinetics, effectively characterizes the Langmuir-type adsorption–desorption process [40,41,42,43,44]. This model considers the inter-phase mass transfer as the rate-limiting step and is commonly used to analyze breakthrough curves, which depict the concentration versus time relationship, as shown in Equation (16) [45].

(16) $$\ln\left(\frac{C_{0}}{C_{t}}-1\right)=\left(\frac{k_{T}^{*}\times q_{e}\times W_{M}}{F}\right)-\left(k_{T}\times C_{0}\times t\right)$$

where $k_{T}^{*}$ is the Thomas model constant (mL/min·mg), and $q_{e}$ is the equilibrium binding capacity in flow mode (mg/g). The values of the constants $k_{T\;}^{*}$ and $q_{e}$ can be determined by calculating the slope and intercept from the linear plot of ln[(*C*_0_*/C_t_*) − 1] against time, *t*.

#### 2.8.2. BDST Model

The BDST model, initially proposed by Bohart and Adams [46], provides an understanding of the adsorption rate, which is influenced by the surface reaction of the adsorbate (AO7) and the residual capacity of the adsorbent. The linear representation of this model is expressed by Equation (17) [40,41,42,43,44,47].

(17) $$t=\left(\frac{Q_{0}\times Z}{C_{0}\times \upsilon }\right)-\left(\frac{1}{k_{BDST}\times C_{0}}\right)\times \ln\left(\frac{C_{0}}{C_{t}}-1\right)$$

The values of the binding capacity of the membrane bed per unit bed volume (*Q*_0_, mg/mL) and the kinetic rate constant (*k_BDST_*, mL/(mg·min)) can be obtained by analyzing the intercept and slope from the linear plot of t against ln[(*C*_0_*/C_t_*) − 1].

### 2.9. Data Analysis

Each experiment was conducted three times, and the results are reported as the mean values. Data analysis was performed with an error margin of less than ±5%.

## 3. Results and Discussion

### 3.1. Nanofiber Membrane Properties

When investigating the adsorption capability of NM-COOH-CEW nanofiber membranes on AO7 dye, enhanced thermal stability was observed, displaying a pattern comparable to the adsorption behavior of TBO by NM-COOH-CEW nanofiber membranes [18]. Detailed information on the physical and chemical properties of the nanofiber membrane can be found in Supplementary Information (Figures S3–S5 and Table S1). Furthermore, the characterization of the nanofiber membrane using scanning electron microscopy (SEM)(H-800, Hitachi Ltd., Tokyo, Japan), thermogravimetric analysis (TGA)(PerkinElmer Co., Waltham, MA, USA), and Fourier transform infrared spectroscopy (FTIR) (TENSOR II, Bruker, Germany) is presented in Figures S3–S5, respectively.

In this study, a thorough characterization of NM-COOH-CEW nanofiber membranes was carried out using various analytical techniques, including FTIR, SEM, and TGA, to examine their structural and morphological properties and their influence on AO7 dye adsorption. FTIR analysis confirmed the presence of important functional groups (-COOH, -NH_2_) on the CEW protein-modified nanofiber membranes, indicating potential binding sites for dye molecules. SEM imaging revealed the surface morphology and structure of the nanofibers, highlighting surface porosity and pore size (see Table S1), which are critical factors affecting dye adsorption capacity. TGA provided insights into thermal stability, crucial for understanding membrane performance under different conditions. By integrating these characterization results, this study aimed to establish correlations between nanofiber properties and AO7 dye adsorption behavior, thereby enhancing the understanding of dye removal mechanisms and membrane performance.

Several parameters significantly affect the adsorption capacity of nanofiber membranes for dyes like AO7. These parameters include the density and types of surface functional groups (-COOH, -NH_2_) on the nanofiber membrane, which influence the affinity and binding interactions with dye molecules. Carboxyl (-COOH) and amino (-NH_2_) groups can facilitate electrostatic interactions and hydrogen bonding with dye molecules, enhancing adsorption capacity. Additionally, the surface area and porosity of the nano-fiber membrane impact the accessible binding sites for dye molecules. Higher surface area and porosity typically correlate with increased adsorption capacity due to more available active sites. The specific results of these characterizations for the nanofiber membranes are detailed in Table S1, providing valuable insights into the structural features that govern dye adsorption behavior and membrane performance.

### 3.2. Optimization of CEW Immobilized onto Acidic Nanofibers

#### Coupling Concentration and Adsorption pH for CEW

A one-step coupling method combined chicken egg white (CEW) with NM-COOH nanofiber membranes at pH 4.75 [18]. This process activated NM-COOH carboxyl groups and linked them with CEW amino groups using EDC/NHS chemistry. A 1:1 molar ratio of -COOH groups to EDC/NHS optimized coupling efficiency and specificity [18,48].

**Figure 1:**
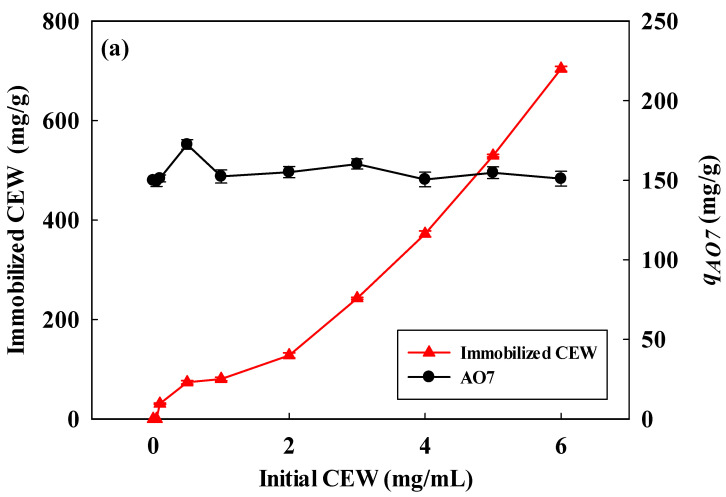

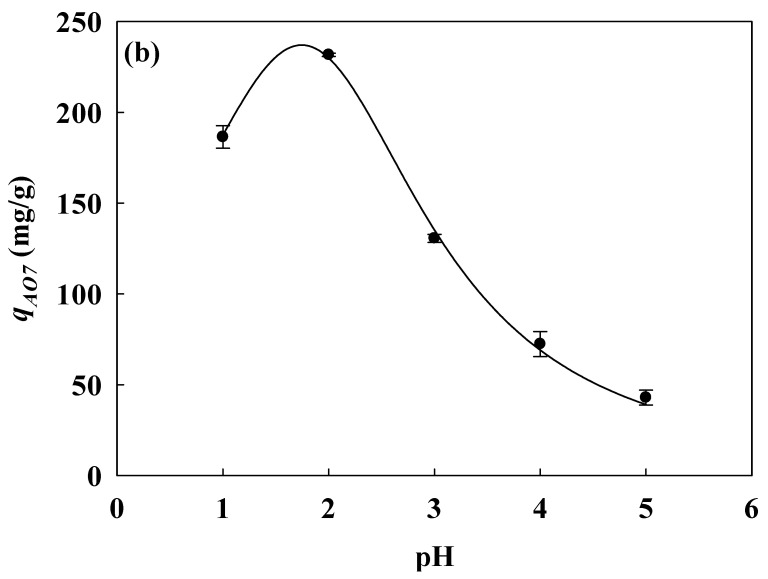
(**a**) Influence of initial CEW concentration (mg/mL) on immobilized CEW protein concentration (mg/g) and AO7 dye adsorption capacity (*q*_AO7_, mg/g). Experimental parameters: ~0.03 g NM-COOH membrane, 0.1 mg/mL–6.0 mg/mL CEW concentration (5 mL, pH 4.75, 3 h, 100 rpm) and (**b**) Influence of buffer pH on AO7 dye adsorption capacity. Experimental parameters: ~0.03 g NM-COOH-CEW nanofiber membrane and 1.0 mg/mL AO7 concentration (5 mL, pH 1–5, 100 rpm). Lines (in Figure 1a) serve as visual aids to illustrate trends among adjacent data points.

In this work, we explored how varying chicken egg white (CEW) concentrations impacted the immobilization density when combined with NM-COOH nanofiber membranes. Increasing CEW concentration from 0.1 to 0.35 mg/mL increased CEW immobilized density from 30.6 mg/g ± 1.2 mg/g to 49.7 mg/g ± 1.0 mg/g (Figure 1a). The highest AO7 dye removal (589 mg/g ± 12 mg/g) was achieved at a CEW concentration of 0.35 mg/mL. However, exceeding this concentration reduced AO7 removal due to steric hindrance from excess immobilized CEW protein, which hindered AO7 access to binding sites. Carboxylic acid groups (NM-COOH) transition to carboxylate groups (-COO^−^) with a negative charge at higher pH levels (above 4–5). CEW proteins typically have an isoelectric point (*pI*) ranging from 5.5 to 6.0 [49]. Below the *pI*, CEW proteins carry a net positive charge due to amino acid protonation. AO7 adsorption by NM-COOH-CEW nanofiber membranes was carried out at pH values below 5 to leverage the positive charge on CEW proteins. The sulfonic acid group (-SO_3_H) in AO7 has a low pKa (often below 1), indicating high acidity. At pH values above 1, the sulfonic acid group is mostly deprotonated (-SO_3_^−^), while at pH values below 1, it remains protonated (-SO_3_H). At very low pH levels like pH 1 and 2, AO7 becomes significantly protonated, changing its overall charge to positive and affecting its solution color and absorption spectra.

In Figure 1b, the CEW-modified membrane shows a positive charge dominated by -NH_3_^+^ groups between pH 1 and pH 5 [18], facilitating interaction with negatively charged AO7 dye through charge–charge interactions. As pH decreased, the adsorption capacity for AO7 increased, peaking at pH 2 (232 mg/g ± 10 mg/g removal capacity). At pH 1, the removal capacity was lower (186 mg/g ± 20 mg/g), but the positive membrane charge still allowed interaction with partially deprotonated dye molecules. Other membrane functional groups likely contributed to enhanced adsorption capacity under different pH conditions, illustrating a complex relationship between dye behavior and membrane charge. Figure S6 displays various color photographs of nanofiber membranes. The NM-COOH-CEW-AO7 nanofiber membrane exhibited a deep orange color after being treated with AO7.

### 3.3. Kinetic Studies

**Figure 2:**
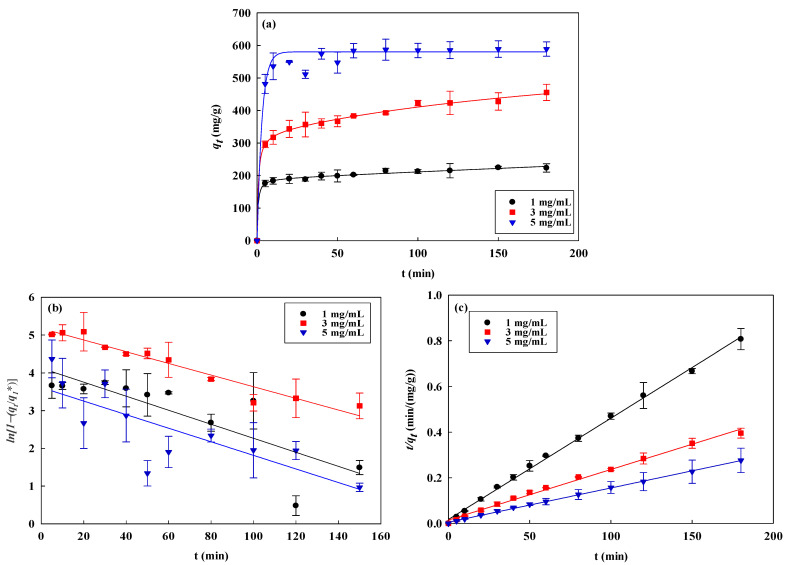

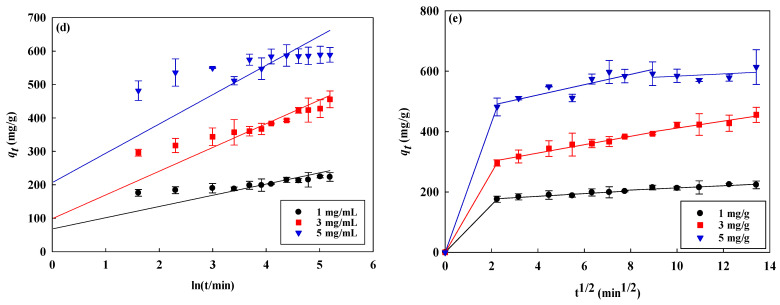
(**a**) Time-dependent AO7 dye removal by NM-COOH-CEW membranes at different concentrations (1.0–5.0 mg AO7/mL), in good agreement with the pseudo-second-order model. Kinetic results fitted by (**b**) the pseudo-first-order kinetic model, (**c**) the pseudo-second-order kinetic model, (**d**) the Elovich model, and (**e**) the intra-membrane diffusion model.

The NM-COOH-CEW nanofiber membrane’s AO7 removal rate was studied across different concentrations (1.0–5.0 mg/mL) and temperatures (298–318 K). Equilibrium was reached within 30 min, indicating rapid AO7 binding to the membrane’s protein sites. At 298 K, the removal capacity increased from 232 mg/g ± 10 mg/g to 573 mg/g ± 25 mg/g as the initial AO7 concentration increased from 1.0 to 5.0 mg/mL, as illustrated in Figure 2a. Higher AO7 concentrations resulted in increased removal capacity due to more significant concentration gradients that drive mass transfer between the aqueous and membrane phases.

**Figure 3:**
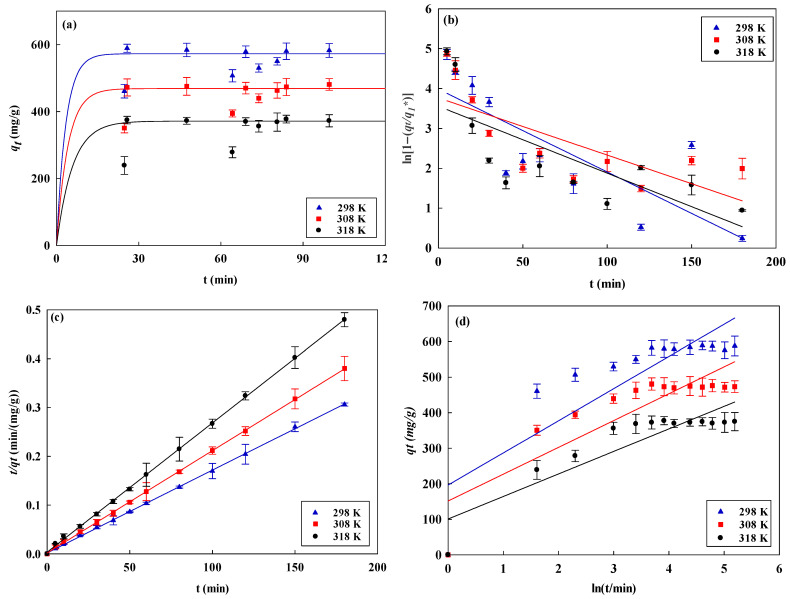

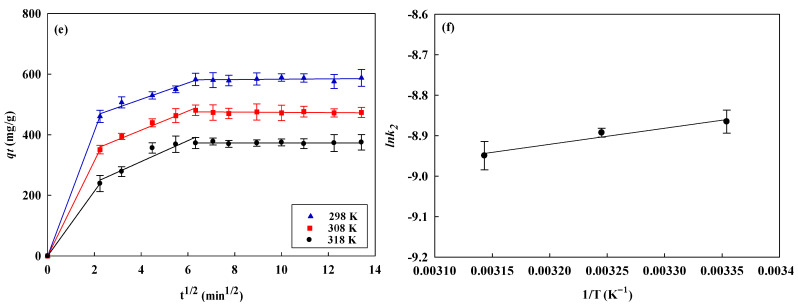
(**a**) Time-dependent AO7 dye removal by NM-COOH-CEW membranes at different temperatures (298–318 K), in good agreement with the pseudo-second-order model. Kinetic results fitted by (**b**) the pseudo-first-order kinetic model, (**c**) the pseudo-second-order kinetic model, (**d**) the Elovich model, (**e**) the intra-membrane diffusion model, and (**f**) the Arrhenius plot of *lnk*_2_ against *1/T* to give the activation energy.

Temperature significantly affected adsorption reactions, causing a reduction in removal rate as temperature increased. For example, at 5.0 mg AO7/mL, the capacity decreased from 589 mg/g ± 12 mg/g to 378 ± 12 mg AO7/g membrane as the temperature increased from 298 K to 318 K, consistent with findings from TBO removal experiments. This thermal sensitivity of membrane responses is highlighted in Figure 3a. The similar behavior observed during TBO removal underscores the consistency of nanofiber membrane responses [18]. This phenomenon aligns with adsorption theory, where higher temperatures lead to decreased adsorption and potential desorption of previously adsorbed molecules, following Le-Chatelier’s principle [50,51,52,53,54,55,56].

We applied four kinetic models (pseudo-first order, pseudo-second order, Elovich, and intra-membrane diffusion) to analyze experimental results at different concentrations and temperatures, as depicted in Figure 2b–e and Figure 3b–e. The pseudo-second-order model provided an excellent fit (*R*^2^ > 0.99), as shown in Table 1. The rate constants (*k*_2_) increased from 6.02 × 10^−5^ to 1.41 × 10^−4^ mg AO7/min·g for AO7 concentrations ranging from 1 mg/mL to 5 mg/mL, and from 1.41 × 10^−4^ to 1.30 × 10^−4^ mg AO7/min·g at temperatures of 298 K to 318 K, respectively. These results confirm that the pseudo-second-order kinetic model is suitable for describing AO7 removal by nanofiber membranes, offering high accuracy and a strong correlation with experimental values.

The pseudo-second-order kinetic model suggests that AO7 chemisorbs onto the NM-COOH-CEW membrane, forming strong chemical bonds with the adsorber surface. Initially, AO7 molecules interact with active sites on the surface, forming covalent or ionic bonds. As more AO7 molecules bind, the adsorber becomes saturated.

To determine the activation energy (*E_a_*) for this reaction, we analyzed the kinetic rate constant (*k*_2_) at temperatures ranging from 298 K to 318 K using the Arrhenius equation and the pseudo-second-order kinetic model [57]. This analysis quantitatively explores the relationship between temperature and reaction rate.

(18) k2=Ae−EaRT

The nanofiber membrane demonstrates a −3.31 kJ/mol activation energy (*E_a_*) for AO7 removal, as depicted in Figure 3f on an Arrhenius plot, signifying barrierless reactions at higher temperatures. AO7 adsorption onto NM-COOH-CEW nanofibers involves stages where molecules migrate to the surface, interact with functional groups, diffuse into pores, and traverse the membrane structure [25,28]. Understanding these stages helps control mass transfer and adsorption on the membrane surface.

### 3.4. Isotherm Studies

**Figure 4:**
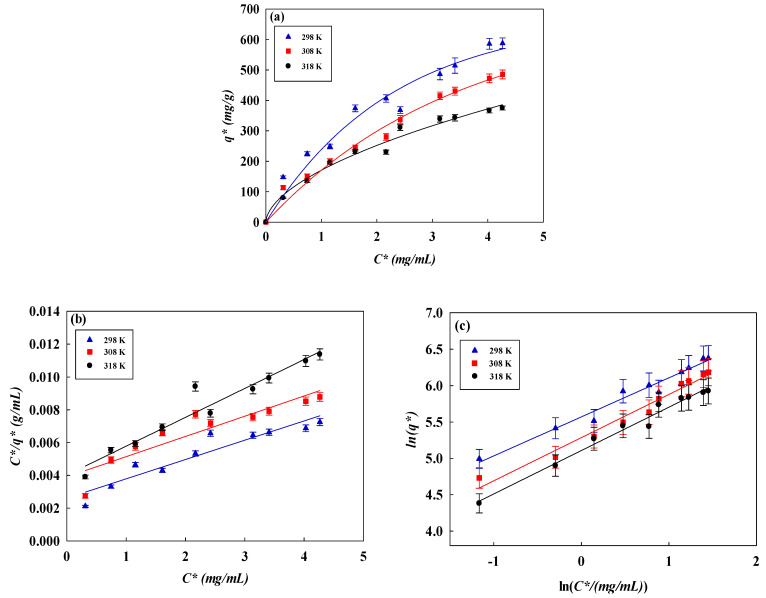

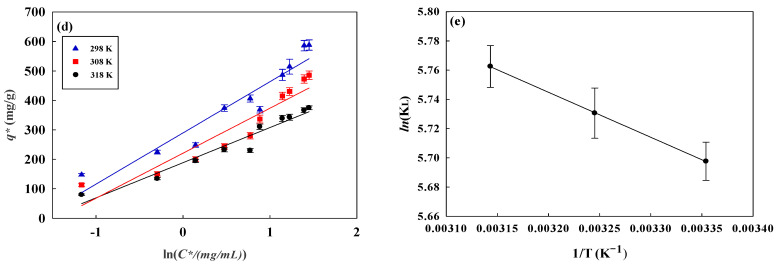
(**a**) Equilibrium studies for the removal of AO7 dye by the NM-COOH-CEW nanofiber membrane under different temperatures (298–318 K), consistent with the Freundlich model. Equilibrium results fitted by (**b**) the Langmuir model, (**c**) the Freundlich model, (**d**) the Temkin model, and (**e**) the van’t Hoff plot of ln (*K_L_*) versus 1/T to give thermodynamic parameters.

We used three equilibrium isotherm models (Langmuir, Freundlich, and Temkin) to analyze AO7 dye adsorption on NM-COOH-CEW nanofiber membranes, as shown in Figure 4a–d [17,23,53]. The Freundlich model (*R*^2^ > 0.99) was most suitable, indicating favorable adsorption (*n_F_* > 1) with multilayer adsorption on a heterogeneous surface. The Langmuir and Temkin models did not fit well (*R*^2^ = 0.84–0.94 and *R*^2^ = 0.89–0.73, respectively). The Langmuir constant (*K_L_*) assesses AO7-NM-COOH-CEW affinity, while higher *K_F_* in the Freundlich model indicates greater adsorption capacity and higher *n_F_* suggests stronger adsorption intensity and surface heterogeneity. The parameters of all isotherms are listed in Table 2.

Both NM-COOH-CS-CEW and NM-COOH-CEW followed the Freundlich model, with NM-COOH-CS-CEW showing higher *K_F_* (286.7 mg/g) and *n_F_* (7.12) compared to NM-COOH-CEW (262.7 mg/g, 1.86). Despite NM-COOH-CEW having a higher adsorption capacity (589 mg/g ± 12 mg/g) than NM-COOH-CS-CEW (329.5 mg/g), this suggests more available binding sites despite weaker individual binding. AO7-NM-COOH-CEW affinity is influenced by factors like hydrogen bonding, electrostatic interactions, surface chemistry, large surface area, high porosity, and selective adsorption based on charge, size, and chemical structure.

The determination of the best-fit isotherm curve models relied on the coefficient of determination (*R*^2^), as shown in Table 2. The Freundlich isotherm model (*R*^2^ > 0.99) represented AO7 dye removal well. The linear plot for the Freundlich isotherm model yields intercept (*K_F_*) values ranging from 262.697 to 165.174 and slope (*n_F_*) values ranging from 1.862 to 1.676 at temperatures from 298 to 318 K. With *n_F_* greater than 1, it indicates favorable adsorption of dye by the membrane adsorber. However, the equilibrium adsorption data did not follow the Langmuir model (*R*^2^ = 0.84–0.94) and the Temkin model (*R*^2^ = 0.89–0.73). High adsorption capacity despite lower binding affinity suggests an abundance of binding sites due to surface heterogeneity, allowing for multilayer adsorption. The porous structure and high surface area of the adsorbent provide numerous binding sites, enhancing adsorption capacity. AO7-NM-COOH-CEW affinity is influenced by specific surface chemistry, large surface area, high porosity, and selective adsorption based on charge, size, and chemical structure.

### 3.5. Thermodynamic Parameters

To ascertain the spontaneity of the AO7 removal process, the thermodynamic parameters of the removal process can be computed under varying temperature conditions (e.g., 298 K, 308 K, and 318 K) using the van’t Hoff Equation (8) [54].

The Gibbs free energy (Δ*G*°) can be calculated using Equation (19), which is as follows:

(19) $$\Delta G^{{}^{\circ}}=\Delta H^{{}^{\circ}}-T\Delta S^{{}^{\circ}}=-RTln\left(K_{L}\right)$$

(20) $$ln\left(K_{L}\right)=\left(-\frac{∆H^{{}^{\circ}}}{R}\right)\frac{1}{T}+\left({\frac{∆S}{R}}^{{}^{\circ}}\right)$$

In plotting ln(*K_L_*) versus 1/T, as shown in Figure 4e, the linear slope and intercept represent −Δ*H*°*/R* and Δ*S*°*/R*, respectively. The calculated results of thermodynamic parameters are listed in Table 3.

Δ*S*° > 0 reflects the increased randomness of the solid–liquid interface during the removal of AO7 on the nanofiber membrane. Additionally, Δ*H*° > 0 indicates that the removal process is an endothermic process. Δ*G*° < 0 suggests that the removal of AO7 by the membrane is a spontaneous process. Moreover, the Δ*G*° value decreased with increasing temperature, implying that higher temperatures are advantageous for the membrane removal of AO7. This can be explained by the fact that an increase in temperature increased the probability of the binding site being in contact with the AO7 dye molecule. According to the thermodynamic theory of binding energy [55,56], in the case in which Δ*H*° > 0 and Δ*S*° > 0, hydrophobic interaction prevailing in the binding process predominantly influences the interaction between the AO7 dye and immobilized CEW proteins.

In our prior investigation concerning the removal of TBO dye by the identical NM-COOH-CEW nanofiber membrane, Δ*H*° < 0 and Δ*S*° < 0 indicated that van der Waals forces and hydrogen bonds have a predominant role in the interaction between TBO dye and immobilized CEW proteins [18]. Consequently, conformational alterations in immobilized CEW proteins following binding with various dye molecule structures may ensue. This phenomenon also influences the varied thermodynamic parameter results.

### 3.6. Desorption Studies

#### 3.6.1. Effect of Organic Solvents

The organic solvents utilized in the elution process can diminish liquid polarity and mitigate hydrophobic interactions between dye molecules and immobilized CEW proteins. Thus, the elution efficiency (*EE*, %) of AO7 was investigated across various concentrations of organic solvents (e.g., 10–50% *v/v* solutions of EG, ethanol, or glycerol). The findings indicated that the highest elution efficiency (*EE*, %) values were observed to be 28.31%, 42.25%, and 38.03% for 25% ethanol, 50% EG and 10% glycerol, respectively, as depicted in Figure 4a.

#### 3.6.2. Effect of Liquid Ionic Strength and Salts

In these experiments, to analyze the effect of salts on the elution efficiency (*EE*, %) of AO7 dye, different types of salts and salt concentrations (0.25–1.0 M NaCl, (NH_4_)_2_SO_4_, and KSCN) were used to elute for the AO7 adsorbed by the nanofiber membrane. The elution results showed that the EE value with 0.25–1.0 M NaCl and with 0.25–1.0 M (NH_4_)_2_SO_4_ was 27.61–21.35% and 29.94–21.36%, respectively, as shown in Figure 4b. Similar elution values were observed for these two salts. However, the EE value with 0.25–2.0 M KSCN reached the maximum at 0.5 M, approximately 55.35%. When the concentration was higher than 0.5 M, the *EE* value significantly decreased to 20.81% with 2.0 M, indicating that some hydrophobic force was present. Hence, hydrophilic and ionic interactions were present between AO7 and immobilized CEW. A higher *EE* (%) value was observed at a lower concentration of salts. Compared to the EE value with organic solvents as an eluent, the elution performance is significantly influenced by the ionic strength of the process liquids, as electrostatic interactions may contribute to binding mechanisms.

#### 3.6.3. Effect of Composite Eluents

When employing a combination of organic solvents and salts as eluents, it was observed that AO7 adsorbed by the nanofiber membrane could be eluted from 22.23% to 78.62% using 0.5 M KSCN in 25–100% EG % solution, as illustrated in Figure 4c. These results suggest that the combined effects of ionic interactions and hydrogen bonds may be implicated in the interaction between AO7 and CEW proteins.

#### 3.6.4. Regeneration

The regenerated CEW membrane was used to remove AO7 under identical conditions (5.0 mg/mL AO7 at pH 2 and 298 K), as shown in Figure 4d. It was noted that the removal capacity after regeneration with composite solvents was lower than that of the virgin membrane, but the capacity of both membranes for AO7 was similar. In some cases, the removal capacity of the regenerated nanofiber membrane exceeded that of the virgin nanofiber membrane significantly when using 1.0–2.0 M KSCN or 75–100% EG as a solvent. These results indicate that the removal capacity of the nanofiber membrane is greatly influenced by the residual AO7 molecules following the elution step. This phenomenon may stem from conformational changes in the binding site of the nanofiber surface structure. Residual AO7 left after regeneration can increase adsorption capacity during subsequent adsorption, which is influenced by various factors. Partial desorption during regeneration may displace AO7 from less favorable sites, exposing them to fresh binding. Regeneration can also reconfigure active binding sites, increase membrane affinity through a memory effect, and induce structural changes like surface alterations that improve binding site accessibility. Overall, these factors contribute to the membrane’s improved performance in adsorbing AO7 across multiple cycles.

### 3.7. Removal of Dyes in Flow Process

**Figure 5:**
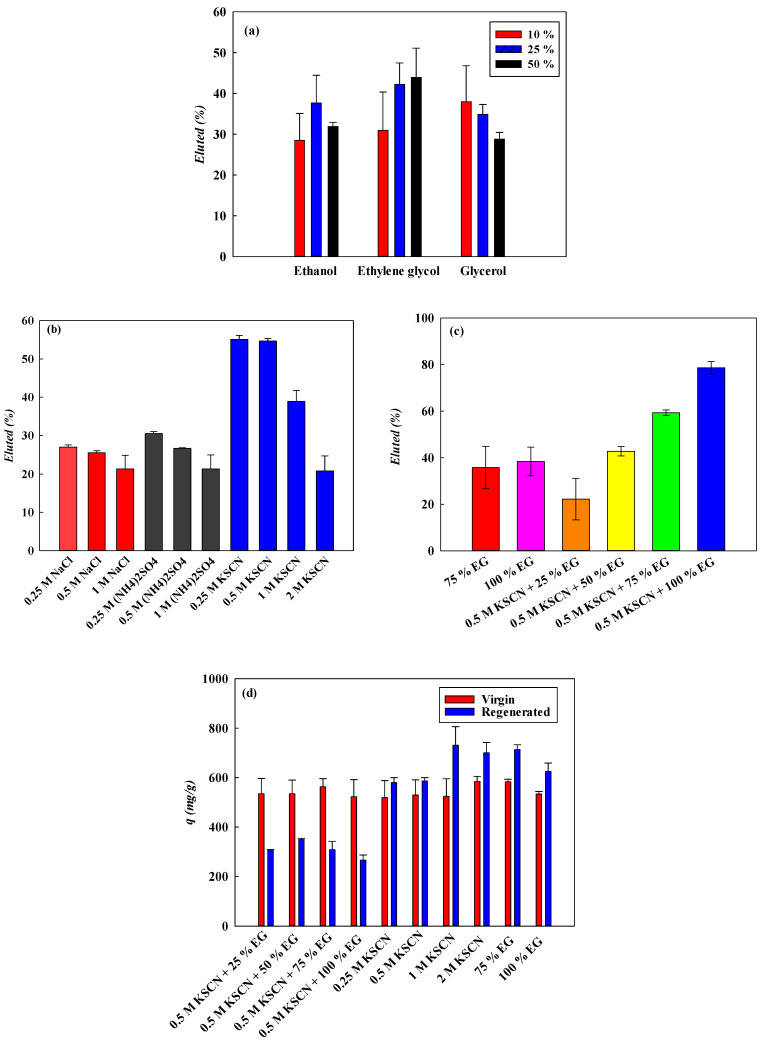
Elution efficiency (*EE*, %) of the adsorbed AO7 dye from NM-COOH-CEW nanofiber membrane by using different (**a**) organic solvents, (**b**) salts, and (**c**) combined solvents; (**d**) comparison of removal capacity of AO7 by using virgin and regenerated NM-COOH-CEW nanofiber membrane under different eluents.

In a previous study, the removal of TBO dye by the NM-COOH-CEW nanofiber membrane was investigated [18]. In this study, experiments were conducted to compare the dynamic removal behavior of two dyes, AO7 and TBO, using the same NM-COOH-CEW nanofiber membrane. In the flow system, AO7 and TBO dyes were individually carried out at pH 2 and pH 10, with the same dye concentration of 1.0 mg/mL and flow rate of 1.0 mL/min. The breakthrough curves for both dyes are depicted in Figure 5, and the parameters calculated from the breakthrough curves are presented in Table 4. It was observed that the values of *V_b_* and *BV* for AO7 dye were lower than those for TBO, suggesting that the adsorption performance of the NM-COOH-CEW nanofiber membrane for TBO was higher than for AO7. However, the values of *HMTZ* and *MAER* for TBO were smaller than those for AO7. This indicates that the removal performance is higher when the values of *HMTZ* and *MAER* are smaller. Hence, the dynamic adsorption performance of TBO dye was superior to that of AO7 dye. From the results of the breakthrough curve parameters (*V_b_*, *BV*, *HMTZ*, and *MAER*), it can be expected that the DBC for TBO dye (230.0 mg/g) would be higher than that for AO7 dye (291.3 mg/g). Similarly, the EBC for TBO dye (385.1 mg/g ± 9.7 mg/g) would be higher than that for AO7 dye (318 mg/g ± 10 mg/g). The *MBU* (%) of TBO dye would be higher than that of AO7 dye. However, the same productivity of 66.67 (mg/g·min) for both dyes was observed under the same operating conditions.

Based on the batch isothermal experiment at 1 mg/mL of AO7 dye, the binding capacity was 232 mg/g ± 10 mg/g, as described in Section 3.2. The DBC for AO7 in a flow process was 230.0 mg/g, corresponding to 95.32% of the equilibrium binding capacity in batch mode. However, the EBC of 318 mg/g ± 10 mg/g was much higher than the equilibrium binding capacity of 230.0 mg/g under the same concentration of AO7 dye. Hence, multilayer binding behavior for AO7 on the NM-COOH-CEW would have occurred in the flow system.

### 3.8. Breakthrough Curve Modeling

**Figure 6:**
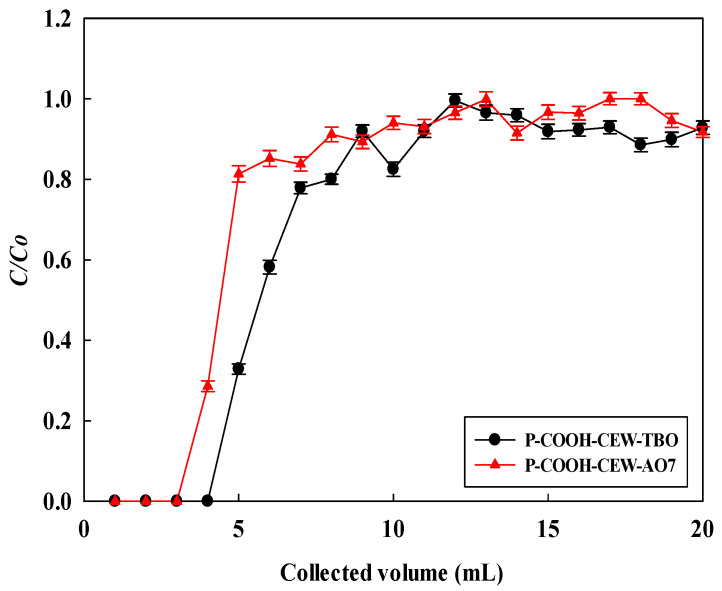
Breakthrough curves for the AO7 and TBO dye adsorption by NM-COOH-CEW nanofiber membrane. A single piece of NM-COOH-CEW nanofiber membrane weighing 0.015 g (with an effective surface area of 3.7 cm^2^) was used for loading AO7 dye at a concentration of 1.0 mg/mL (at pH 2) and TBO dye at 1.0 mg/mL (at pH 10). The flow rate during the experiment was maintained at 1.0 mL/min, resulting in a flux of 0.27 cm/min.

As indicated in Table 5, a significant difference exists between the experimental and predicted results of the adsorption breakthrough curves for the two dyes (AO7 and TBO, see Figure 6). The fitting regression coefficient (*R*^2^) ranged from 0.71 to 0.89, and the error percentage (*E*, %) between the calculated value (*q_e_*) from the experimental results and the Thomas model varied from 33.45% to 51.16%. Consequently, this model proves unsuitable for accurately describing the dynamic adsorption behavior of AO7 and TBO dyes.

The breakthrough parameters for fitting the BDST model are presented in Table 5. The findings indicate that the Qo values predicted by the BDST model closely align with the experimental values. The *R*^2^ values ranged between 0.89 and 0.91, and the error percentage (*E*, %) values ranged between 0.46% and 3.55%. Hence, the BDST model proved acceptable for accurately describing the dynamic adsorption behavior of the two dyes.

### 3.9. Remarks on the Comparison with Other Adsorbers for AO7 Dye

The results demonstrate significantly higher AO7 removal efficiency compared to other adsorbers in the literature (Table 6) [22,51,52,53,54,55,57,58]. Waste CEW solution from the lab was used to create a CEW nanofiber membrane on a weak acidic NM-COOH nanofiber, achieving maximum AO7 removal efficiencies up to 589 mg/g ± 12 mg/g (298 K). With metal-chelating functional groups like His and Cys amino acids in the CEW protein nanofiber membrane, it could also be effective for treating heavy metal wastewater.

The developed CEW membrane effectively handles positively charged dyes at high pH levels (e.g., TBO at pH 10) and efficiently removes negatively charged dyes (e.g., AO7 at pH 2). CEW protein nanofiber membranes are anticipated to address both positively and negatively charged dyes across a wide pH range, enhancing practical use in dye wastewater treatment. A comparison of AO7 adsorption on different functional nanofiber membranes (NM-COOH-CS-CEW) in a previous study [22] with NM-COOH-CEW in this study highlights how nanofiber matrix selection affects the adsorption behavior.

Surface chemistry, functional groups, and structural properties significantly influence dye adsorption characteristics, providing insights into substrate composition’s role in dye removal. This study extends AO7 adsorption investigation to batch and flow systems using NM-COOH-CEW nanofiber membranes, offering insights into kinetics, mass transfer, and efficiency under varying flow conditions.

Both studies likely used similar methods for adsorption investigations, including parameters like dye concentration, pH, contact time, and characterization techniques. However, differences in nanofiber properties and adsorption conditions could lead to variations in kinetics, equilibrium behavior, and capacities. Understanding these distinctions is crucial for interpreting and comparing results effectively, highlighting how nanofiber composition affects AO7 dye adsorption. These findings have important implications for applications such as wastewater treatment, dye removal, and environmental remediation. Understanding AO7 adsorption on different nanofiber membranes guides the design of tailored adsorption processes for specific dye pollutants, advancing water purification and environmental protection technologies. The higher adsorption capacity of NM-COOH-CEW nanofiber membranes compared to NM-COOH-CS-CEW nanofiber membranes containing chitosan for AO7 and TBO dyes can be attributed to several factors [17,18,22]. Firstly, chitosan in NM-COOH-CS-CEW alters surface chemistry, potentially competing with AO7 and TBO for adsorption sites, reducing available capacity. Secondly, electrostatic interactions between chitosan’s amino groups and negatively charged dye molecules may enhance adsorption but hinder desorption, leading to decreased overall adsorption capacity. Additionally, chitosan inclusion could alter pore structure or reduce active site accessibility, impeding dye diffusion and adsorption. Lastly, chitosan’s binding affinity for certain dyes might attract dye molecules preferentially over CEW, reducing effective surface area for CEW-mediated adsorption and overall adsorption capacity for AO7 and TBO dyes.

## 4. Conclusions

In this study, we explored the efficacy of three-dimensional CEW protein coating on NM-COOH membranes for dye removal. Our results showed that NM-COOH-CEW protein-modified nanofiber membranes effectively removed positively charged TBO dye (pH 10) and negatively charged AO7 dye (pH 2) from water, primarily through electrostatic forces. AO7 adsorption aligned with the Freundlich model, while TBO fit the Langmuir model. Both dyes exhibited pseudo-second-order kinetics, with maximum removal rates achieved within 30 min, reaching 589 mg/g ± 12 mg/g for AO7 and 546.2 mg/g ± 9.9 mg/g for TBO. However, efficient regeneration using EG or KSCN eluent showed limitations. Nonetheless, the membrane remains promising for dye removal from contaminated water, contributing to sustainable water treatment techniques. Treating AO7 dye in real wastewater poses challenges due to matrix complexity, competing adsorbates, and optimization needs. Wastewater’s diverse compounds influence adsorption behavior, affecting kinetics and capacity. Despite the challenges involved, investigating AO7 treatment in actual wastewater settings offers valuable practical insights into the applicability of NM-COOH-CEW, thereby contributing to its environmental impact and sustainability in water treatment. This aspect will be further explored in future research.

## Figures and Tables

**Table 1:** Comparison of calculating kinetic parameters by different kinetic models and experimental values for the removal of AO7 dye by the NM-COOH-CEW nanofiber membranes at different operating conditions.

Kinetic Models	AO7 Dye (mg/mL)			Temperature (K)		
	1	3	5	298	308	318
q*_,exp_ (mg/g)	232 ± 10	438 ± 15	589 ± 12	589 ± 12	481 ± 17	378 ± 12
Pseudo-first order						
*k*_1_	1.86 × 10^−2^	1.54 × 10^−2^	1.19 × 10^−2^	1.19 × 10^−2^	1.42 × 10^−2^	1.18 × 10^−1^
*R* ^2^	0.6410	0.8644	0.6366	0.6366	0.5475	0.5584
Pseudo-second order						
*k*_2_ (g/mg·min)	6.02 × 10^−5^	1.10 × 10^−5^	1.41 × 10^−4^	1.41 × 10^−4^	1.38 × 10^−4^	1.30 × 10^−4^
q*_,cal_ (mg/g)	225.2	451.5	589.3	589.3	477.5	377.5
*R* ^2^	0.9939	0.9901	0.9996	0.9996	0.9998	0.9996
Elovich						
α	255.21	285.22	924.62	791.94	560.20	310.11
*β*	2.98 × 10^−2^	1.41 × 10^−2^	1.14 × 10^−2^	1.10 × 10^−2^	1.32 × 10^−2^	1.58 × 10^−2^
*R* ^2^	0.7304	0.8419	0.6728	0.7165	0.7381	0.7917
Intra-membrane diffusion						
*k_i_*_2_	10.57	23.49	26.42	27.11	22.56	19.23
*R* ^2^	0.5216	0.6624	0.4391	0.4622	0.4732	0.5228

**Table 2:** Thermodynamic parameters calculated by isotherm models by the NM-COOH-CEW nanofiber membrane for AO7 dye removal.

Temperature (K)	*q_exp_* (mg/g)	Langmuir			Freundlich			Temkin		
		*q_max_* (mg/g)	*K_L_*, (mL/mg)	*R* ^2^	*n_F_*	*K_F_* (mg/g)	*R* ^2^	*b* (J/mol)	*K_T_* (mL/mg)	*R* ^2^
298	589 ± 12	848.2	2.21	0.8895	1.86	262.7	0.9719	14.26	5.30	0.9076
308	481 ± 17	809.2	3.02	0.8414	1.68	197.4	0.9734	16.770	4.25	0.8907
318	378 ± 12	569.2	2.29	0.9389	1.68	165.2	0.9810	22.16	4.860	0.9466

**Table 3:** Equilibrium isotherm parameters for AO7 dye capture by NM-COOH-CEW nanofiber membrane.

Temperature (K)	Δ*G*° kJ/mol	Δ*H*° kJ/mol	Δ*S*° J/(mol·K)
298	−1.97	2.56	15.20
308	−2.83		16.53
318	−2.19		14.94

**Table 4:** Breakthrough curve parameter analysis for the AO7 and TBO dye removal by using NM-COOH-CEW nanofiber membrane.

Dye	Operating Conditions				Breakthrough Curve Parameters									
	Z (cm)	pH	C_o_ (mg/mL)	F (mL/min)	*t*_10*%*_	*t*_90*%*_	*V_b_* (mL)	*BV*	*HMTZ* *(*μm*)*	*MAER* (× 10^−3^)	*DBC* (mg/g)	*EBC* (mg/g)	*MBU* (%)	Productivity (*P*) (mg/min·g)
AO7	115	2	1	1	3.45	7.91	3.45	81.08	64.84	4.35	230.0	318 ± 10	72.33	66.67
TBO	115	10	1	1	4.37	8.96	8.69	102.70	57.17	3.43	291.3	385.1 ± 9.7	75.65	66.67

**Table 5:** Modeling of the dynamic removal parameters for the AO7 and TBO dye adsorption by NM-COOH-CEW nanofiber membrane.

Dye	Operating Conditions				Thomas Model					BDST Model				
	Z (μm)	pH	C_o_ (mg/mL)	F (mL/min)	$k_{T}^{*}$	*q_e_* (cal)	*q_e_* (exp*)*	*R* ^2^	*E* (%)	*k_BDST_*	*N_o_* (cal)	*N_o_* (exp)	*R* ^2^	*E* (%)
AO7	115	2	1	1	0.77	210.4	318 ± 10	0.7134	51.16	2.10	394.9	408.9 ± 7.5	0.9114	3.55
TBO	115	10	1	1	1.14	288.6	385.1 ± 9.7	0.8874	33.45	1.29	547.3	549.7 ± 8.2	0.8874	0.46

**Table 6:** Comparison of adsorption capacity of different adsorbers for AO7 dye.

Type of Adsorbent	*q_max_* (mg/g)	*q_max_* (μmol/g)	References
NM-COOH-CEW Nanofiber membrane	589 ± 12	1681	This work
NM-COOH-CS-CEW Nanofiber membrane	307	876	[22]
Magnetic graphene/chitosan	43	122	[58]
Bottom ash	4	12	[57]
Canola stalk	25	72	[51]
*Azolla rongpong*	77	220	[52]
Spent brewery grains	30	87	[53]
Untreated sugarcane bagasse	28	80	[54]
Beech wood sawdust	5	14	[55]

## Data Availability

Dataset available on request from the authors.

## Supplementary Materials

The following supporting information can be downloaded at: https://www.mdpi.com/article/10.3390/membranes14060128/s1, Figure S1: Chemical structure of TBO and AO7 dyes; Figure S2: Schematic structure of NM-COOH-CEW-AO7 nanofiber membrane; Figure S3: FTIR spectra for PAN (red), NM-COOH (green), NM-COOH-CEW (blue) nanofiber membranes, and AO7 adsorbed on NM-COOH-CEW (black); Figure S4: Scanning electron microscopy SEM images of (a) PAN, (b) NM-COOH, (c) NM-COOH-CEW, (d) NM-COOH-CEW nanofiber membranes, and (e) (f) AO7 dye adsorbed on the NM-COOH-CEW nanofiber membranes; Figure S5: TGA curves of PAN (black), NM-COOH (red), NM-COOH-CEW (blue) nanofiber membranes, and AO7 adsorbed on NM-COOH-CEW (green); Figure S6: Photos of nanofiber membranes with different colors; Table S1: Physical properties of the PAN-modified nanofiber membranes.

## Nomenclature

*A*	Arrhenius constant
*A_M_*	Membrane effective area (cm^2^)
AO7	Acid orange 7 dye
*BV*	Membrane bed volume
*BDST*	Bed depth service time
*b*	Temkin constant (J/mol)
*C*_0_	Initial AO7 dye concentration (mg/mL)
*C**	Equilibrium AO7 dye concentration in aqueous solution (mg/mL)
CEW	Chicken egg white
*Ea*	Activation energy for AO7 dye adsorption
*F*	Flow rate (mL/min)
*HMTZ*	Length of the mass transfer zone
*J*	Permeation flux (mL/cm^2^·min)
*k*_1_	Pseudo-first-order kinetic constant (1/min)
*k*_2_	Pseudo-second-order kinetic constant (g/mg·min)
*k_BDST_*	BDST kinetic rate constant (mL/(mg·min).
*ki*	Intra-particle diffusion rate constant
*I*	A constant with its value proportional to the boundary layer (mg/g)
*K_L_*	Langmuir constant related to adsorption intensity (mL/mg)
*K_F_*	Freundlich constant related to adsorption intensity (mg/g)
*K_T_*	Temkin constant related to adsorption intensity (mL/mg)
$k_{T}^{*}$	Thomas model constant (mL/min·mg)
*MAER*	Membrane adsorber exhaustion rate
*MBU*	Membrane bed utilization (%)
*n*	Freundlich empirical constant and related to adsorption intensity
*P*	Productivity (mg/g·min)
*Q_o_*	Binding capacity of the membrane bed per unit bed volume (mg/mL)
*q*	Removal capacity for AO7 dye in batch mode (mg/g)
*q_*_*	Equilibrium removal capacity for AO7 dye in batch mode (mg/g)
*q_max_*	Maximum removal capacity for AO7 dye in batch mode (mg/g)
*q_t_*	Removal capacity for AO7 dye at any given time t in batch mode (mg/g)
*R*	Gas constant (8.314 J/mol/K)
*T*	Absolute temperature (K)
TBO	Toluidine blue O dye
*t*	Adsorption time (min)
*t*_10%_	Time required for 10% CEW breakthrough (min)
*t*_90%_	Time required for 90% CEW breakthrough (min)
*t_b_*	Time required at 10% breakthrough (min)
*V*	Total volume of permeated solution (mL)
*V_b_*	10% breakthrough volume (mL)
*V_M_*	Membrane volume (mL)
*W_M_*	Weight of the membrane adsorber (g)
*Z*	Length of the membrane bed (cm)
Δ*G*°	Changes in the apparent free energy (J/mol)
Δ*H*°	Changes in the apparent enthalpy (J/mol)
Δ*S*°	Changes in the apparent entropy (J/mol·K)
*τ*	Residence time in the membrane (min)
*ε*	Porosity of the membrane (%)
*α*	Initial sorption rate in the Elovich model (mg/g·min)
β	Constant related to the extent of surface coverage and activation energy for chemisorption in the Elovich model (g/mg)
$k_{T}^{*}$	Thomas model constant (mL/min·mg)
*q_e_*	Equilibrium binding capacity in flow mode (mg/g)
*C_t_*	Breakthrough concentration (mg/mL) at time t (min)
*Z*	Membrane bed height (cm)
*Q*_0_	Binding capacity of the membrane bed per unit bed volume (mg/mL)
*υ*	Linear velocity (cm/min)
