# Supplementary material for: The Utilization of Chicken Egg White Waste-Modified Nanofiber Membrane for Anionic Dye Removal in Batch and Flow Systems: Comprehensive Investigations into Equilibrium, Kinetics, and Breakthrough Curve
Source: Membranes (Basel). 2024 Jun 3;14(6):128. doi: 10.3390/membranes14060128 (PMC11205732; doi:10.3390/membranes14060128)
Supplement: Supplementary file 1 [file membranes-14-00128-s001.zip › membranes-2928583-supplementary.pdf]

Supplementary information

The Utilization of Chicken Egg White Waste-Modified Nanofiber Membrane for Anionic Dye Removal in Batch and Flow Systems: Comprehensive Investigations into Equilibrium, Kinetics, and Breakthrough Curve

Yun-Rou Chen [1,#], Dinh Thi Hong Thanh [1,#], Quynh Thi Phuong Tran [2], Bing-Lan Liu [3], Penjit Srinophakun [4], Chen-Yaw Chiu [1], Kuei-Hsiang Chen [1,*] and Yu-Kaung Chang [5,*]

[1] Department of Chemical Engineering, Graduate School of Biochemical Engineering, Ming Chi University of Technology, New Taipei City 243303, Taiwan.
[2] Faculty of Environment and Labour Safety, Ton Duc Thang University, Ho Chi Minh City, Vietnam.
[3] Department of Applied Chemistry, Chaoyang University of Technology, Taichung 413310, Taiwan.
[4] Department of Chemical Engineering, Kasetsart University, 50 Ngamwongwan Road, Chatuchak, Bangkok 10900, Thailand.
[5] Department of Chemical Engineering and Materials Science, Yuan Ze University, Zhongli Dist., Taoyuan City 320315, Taiwan.

*Correspondence:
ykchang@mail.mcut.edu.tw (Y.-K.C); khchen@mail.mcut.edu.tw (K.-H.C)

Fig. S1. Chemical structure of TBO and AO7 dyes.

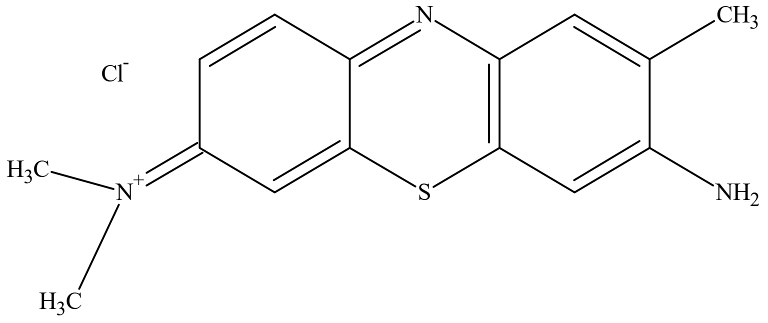

Toluidine blue O (TBO)

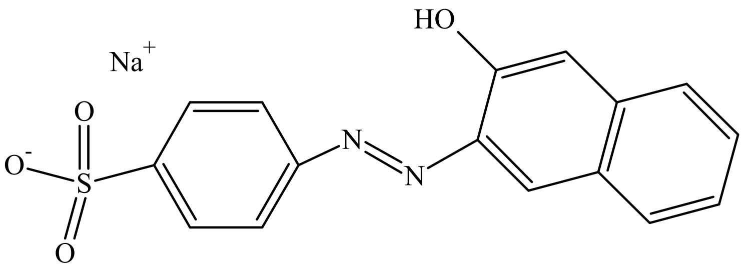

Acid orange 7 (AO7)

Fig. S2. Schematic structure of NM-COOH-CEW-AO7 nanofiber membrane.

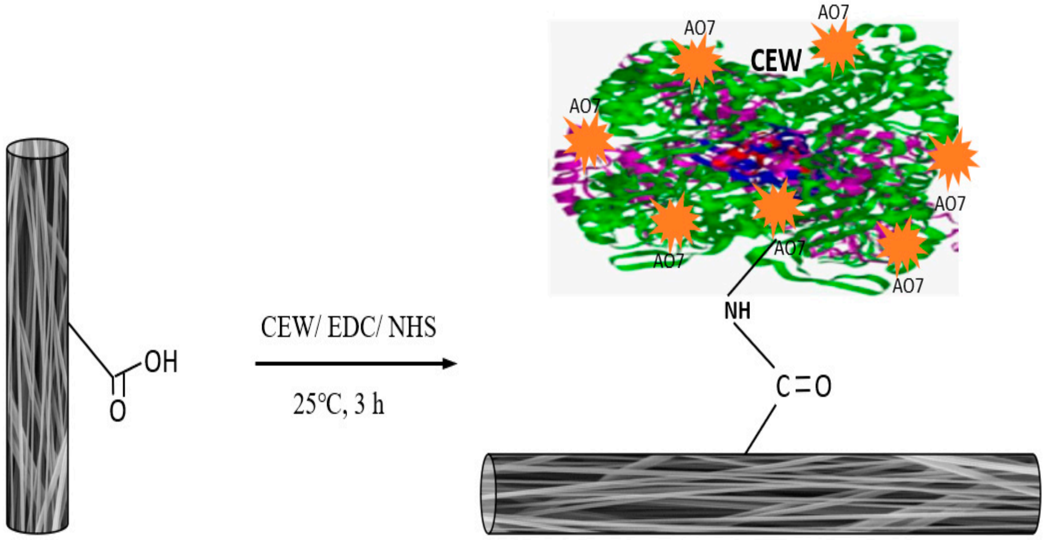

Section S1. Physical property analysis of the nanofiber membranes

Nanofiber membrane preparation was performed according to the protocols established in our previous studies [17-18]. PAN electrospinning, alkaline hydrolysis to produce NM-COOH, and preparation of NM-COOH-CEW nanofibers were extensively investigated in these prior studies. The protein concentration in the solution was assessed using UV-vis spectrophotometry at 595 nm, with BSA serving as the standard protein [17-18]. The immobilization density of CEW (mg/g) was calculated based on the concentration difference before and after immobilization in a fixed aqueous volume (5 mL).

Nanofiber fabrication and surface modification involve a detailed process starting with PAN (polyacrylonitrile) nanofiber fabrication and subsequent conversion to NM-COOH (polyacrylonitrile with carboxylate groups) followed by coupling with chicken egg white (CEW). First, PAN nanofibers are fabricated by preparing a polymer solution containing PAN dissolved in a solvent like dimethylacetamide (DMAc). This solution is then electrospun using an apparatus comprising a syringe pump and a high-voltage power supply to form continuous PAN nanofibers through rapid solvent evaporation on a grounded collector. Next, surface modification to NM-COOH is achieved by chemical treatment, such as NaOH hydrolysis, to introduce carboxylate groups onto the nanofiber surface. The hydrolysis reaction converts nitrile (CN) groups in PAN to carboxylate (COO^-) groups. After washing and neutralization, the NM-COOH-modified PAN nanofibers are coupled with CEW. For coupling, the nanofiber membrane is immersed in a CEW solution and incubated under controlled conditions to facilitate a coupling reaction between carboxyl groups on the membrane surface and amino groups in CEW proteins. This coupling is achieved using EDC/NHS chemistry, ensuring strong attachment and specificity. The molar ratio of -COOH functional groups on the membrane to EDC/NHS was maintained at 1:1. After washing to remove unreacted components, the membrane is dried to ensure stability and efficiency in subsequent adsorption applications.A comprehensive analysis of the physical and chemical properties of these nanofibers was also previously conducted [18]. The functional characteristics of the nanofiber samples were analyzed using Fourier-transform infrared spectroscopy (FTIR, TENSOR II, Bruker, Germany). A scanning electron microscope (SEM, H-800, Hitachi Ltd., Tokyo, Japan) was utilized to study the surface morphology of the samples. Thermogravimetric Analysis (TGA, PerkinElmer Co., Waltham, MA, USA) was employed to investigate the stability of the samples. The distribution of pore size and the mean pore size of nanofiber mats were determined with a permporometer (PMI, USA). The porosity and the specific surface area were measured by a fully automatic density analyzer (AccuPyc 1330 Pycnometer, Micromeritics) and a surface area analyzer (ASAP 2000, Micromeritics), respectively.

Fig. S3. FTIR spectra for PAN (red), NM-COOH (green), NM-COOH-CEW (blue) nanofiber membranes, and AO7 adsorbed on NM-COOH-CEW (black).

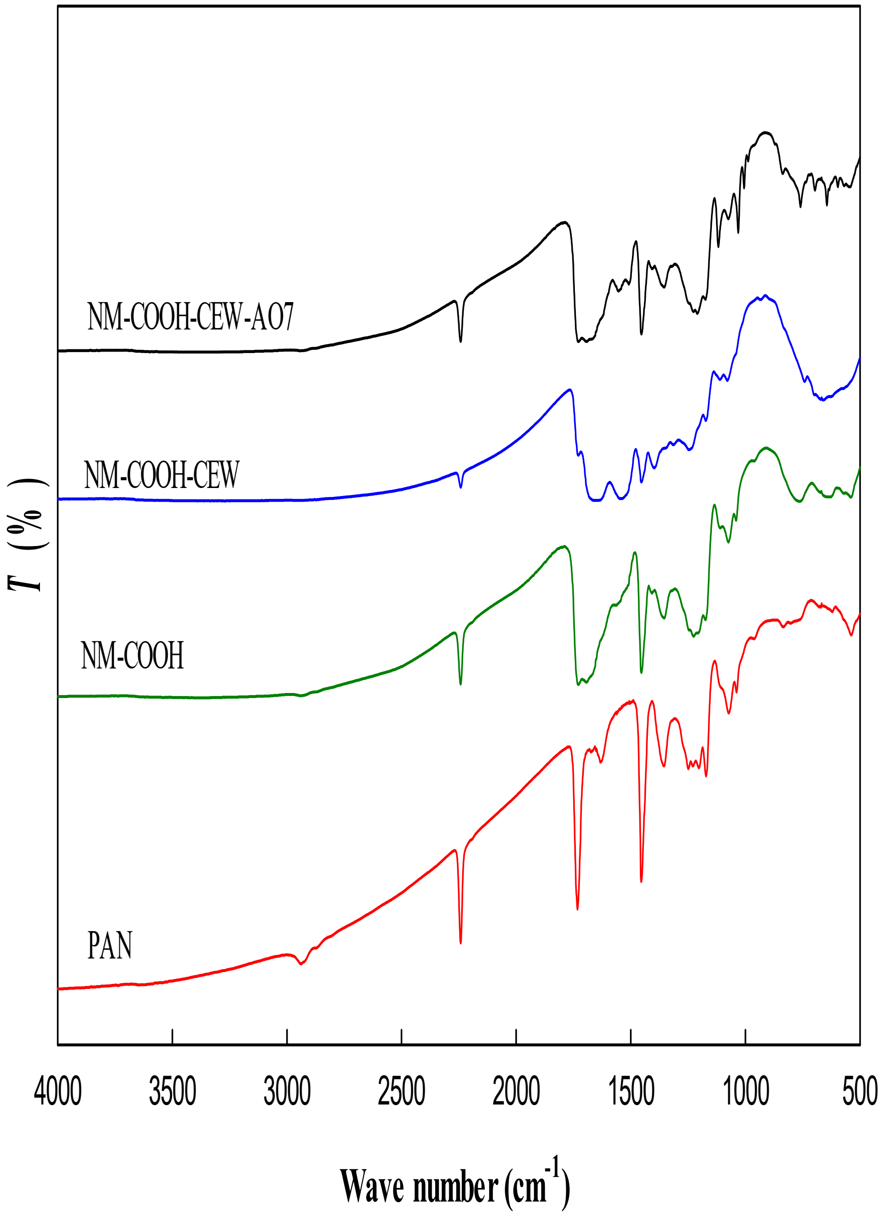

Fig. S4. Scanning electron microscopy SEM images of (a) PAN, (b) NM-COOH, (c) NM-COOH-CEW, (d) NM-COOH-CEW nanofiber membranes, and (e) (f) AO7 dye adsorbed on the NM-COOH-CEW nanofiber membranes.

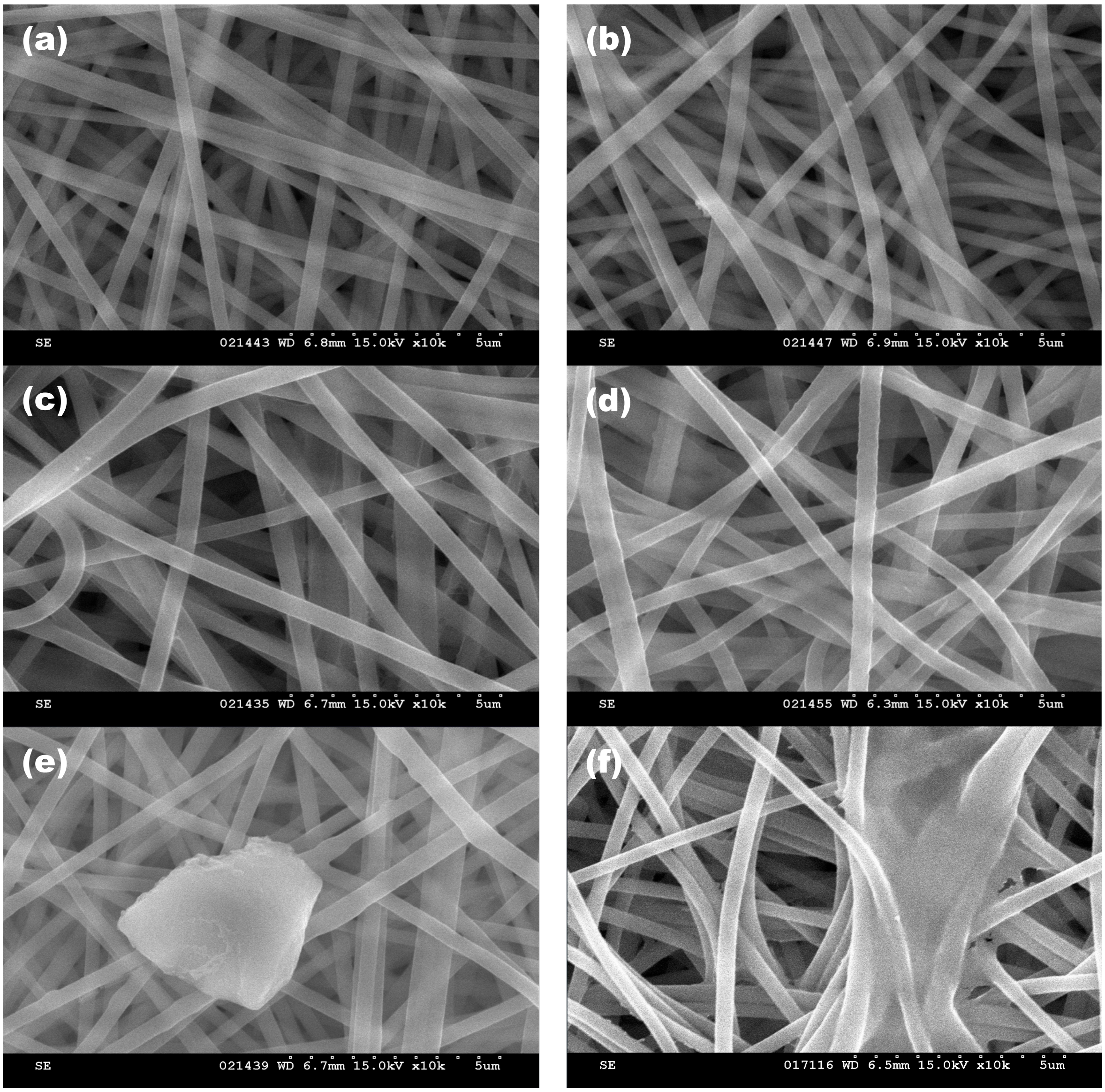

Fig. S5. TGA curves of PAN (black), NM-COOH (red), NM-COOH-CEW (blue) nanofiber membranes, and AO7 adsorbed on NM-COOH-CEW (green).

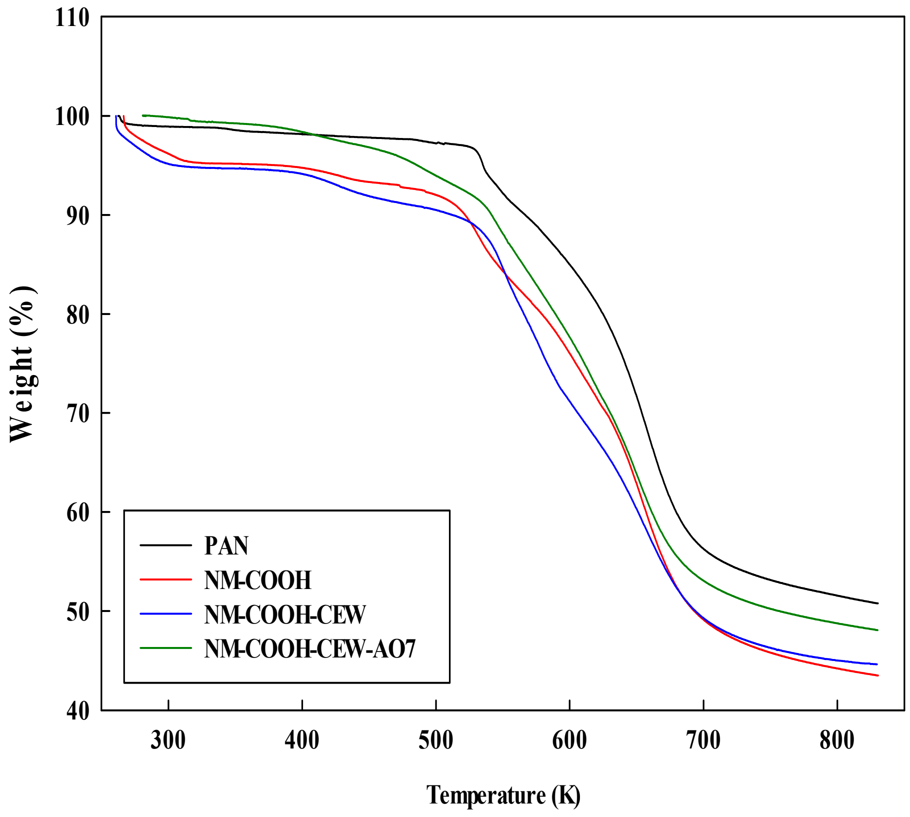

Fig. S6. Photos of nanofiber membranes with different colors.

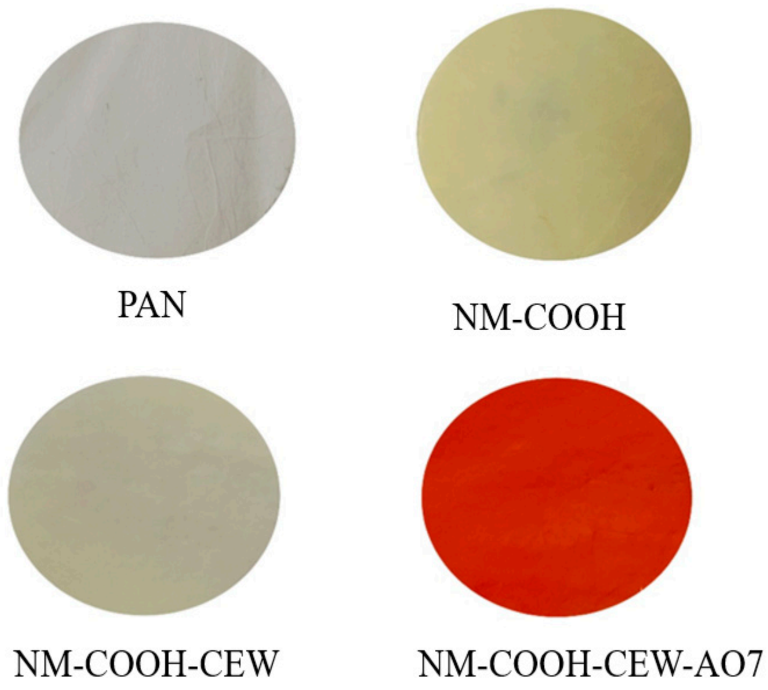

Table S1

Physical properties of the PAN-modified nanofiber membranes.

Membrane	Membrane thickness (μm)	Nanofiber diameter (nm)	Surface porosity (%)	Average pore size (μm)	-COOH (μm)
PAN	232±20	470±20	85.32	0.4406	-
NM-COOH	209±25	452±30	84.26	0.4498	327.37±1.73
NM-COOH-CEW	235±30	495±20	83.65	0.4592	325.46±3.25
